# Editorial: One health, environmental health, global health, and inclusive governance: What can we do?

**DOI:** 10.3389/fpubh.2022.932922

**Published:** 2022-08-05

**Authors:** Ulrich Laaser, Vesna Bjegovic-Mikanovic, Richard Seifman, Flavia Senkubuge, Zeljka Stamenkovic

**Affiliations:** ^1^School of Public Health, University of Bielefeld, Bielefeld, Germany; ^2^School of Public Health, University of Belgrade, Belgrade, Serbia; ^3^American Veterinary Epidemiology Society, Honorary Diplomate, Washington, DC, United States; ^4^University of Pretoria, Pretoria, South Africa

**Keywords:** One Health, environmental health, global health, inclusive governance, bottom-up, top-down

“One Health” is a broad conceptual platform that encompasses a global view of human and animal health, along with the environment, encompassing air, water, soil, and plants. It requires integrating interactions both from bottom-up and top-down perspectives, anchored in an inclusive government (1) and supported by interactive science, i.e., scientific cooperation across disciplines. At the ground level, what is done in practice must influence those governing, leading to more responsive, effective, and inclusive governance and in so doing, much-improved outcomes. Human and veterinary medicine, public health, social sciences, educators, and legislators have to be involved and contribute to what is decided and done at the national and local levels.

An integral element of the One Health concept is the critical importance of addressing the effects of the devastating environmental imbalance of today. This is a major task for our generation and our children, with very limited time left to avoid a planetary catastrophe, aspects of which are already visible. Worldwide, and in many regions and individual countries, current economic, social, and environmental policies are too dangerous and unsustainable. The future state of the environment is, in short, central to One Health's aspirations. The imbalances between developing and developed countries are crucial aspects of this, with many examples that demonstrate this point. For example in this research volume, Streichert et al. discuss the pandemic response, outlining that “lack of opportunities is a commonly reported barrier to involvement globally, with lack of funding the largest barrier in the WHO African region.” Eliakimu and Mans promote the concept of inclusive governance to cope with global inequalities. Differences in COVID-19 vaccine availability showcase the challenge (Figure 1)^1^:

**Figure 1:**
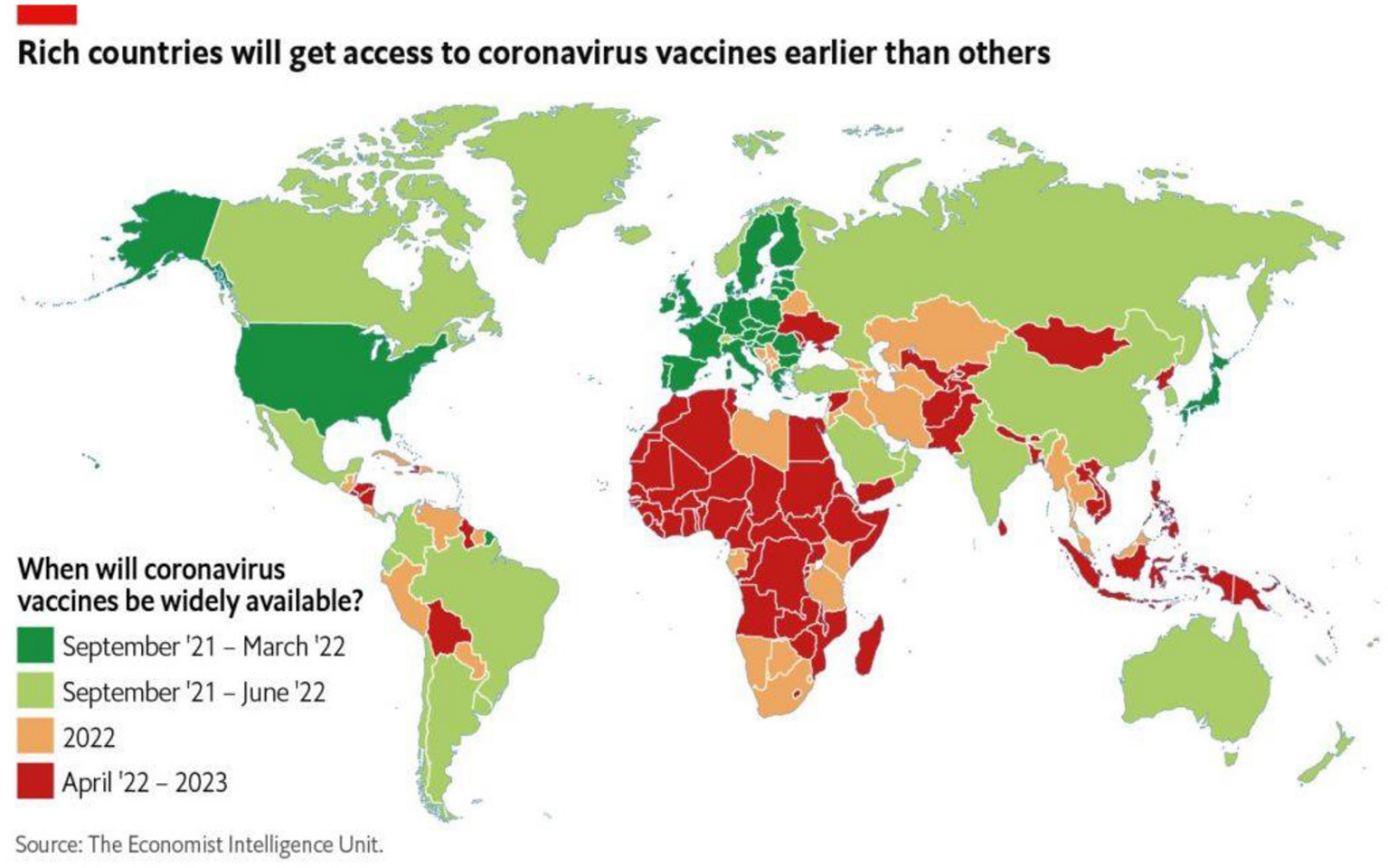
When will coronavirus vaccines be widely available? Source: The Economist Intelligence Unit.

The basic framework for One Health research is captured in THE WORLD IN 2050 INITIATIVE (TWI) (2), which defines six sustainable development pathways:

(1) Education, gender, and inequality.
(2) Health, well-being, and demography.
(3) Energy de-carbonization and sustainable industry.
(4) Sustainable food, land, water, and oceans.
(5) Sustainable cities and communities.
(6) Digital revolution for sustainable development.

These six areas provide the broad framework for the contributions within this Frontiers for Public Health Research Topic. The contributions can be further divided into four groups, of which there are 11 articles published to date.

The *first* group comprises more familiar topics including tobacco control policies in the Indonesian mining industry (Prabandari et al.). Another article in this grouping explores one health perspective regarding diarrhea in Pakistan (Abbasi et al.) while another discusses the quality of life connected with living near a solid waste facility (Phan et al.).

The *second* group deals with the *COVID*-19 pandemic, such as a paper on variations of COVID-19 in the three Palestinian territories, the West-Bank, East-Jerusalem, and Gaza Strip (Abed et al.), covering the spread, risk factors, and interventions. Mahadi discusses the post-COVID AMR threat in LMIC, taking Bangladesh as an example. Another analyses participation in One Health networks as part of a COVID-19 response (Streichert et al.).

The third group covers information and policies. Coming from the same background as Streichert et al., i.e., the One Health Commission, Eliakimu and Mans review inequalities toward inclusive governance. Roopnarine et al. discuss the missing professional perspective of One Health for medical, veterinary, and public health students. LeBlanc et al. underline bioethics and One Health to build reflexive governance. Finally, Yu et al. analyze the effects of environmental information on elderly people in China.

The fourth area deals with satisfaction in life, which is often integrated under the Subjective Well-being (SWB) rubric, as documented here for three countries in the Western Balkans: Montenegro, North Macedonia, and Serbia by Bjegovic-Mikanovic et al. As outlined in this study, Montenegro takes a top position in almost all dimensions.

To achieve significant One Health breakthroughs, there must be diminishing global economic and social disparities between countries and within countries. Even in the most developed countries, underserved populations are substantial in numbers. Their lot has to be improved if we want to follow a civil discourse, both within countries and between them, and at global levels. The unfortunate reality is exemplified during these last two pandemic years by the case of vaccine availability and supporters attacked by the anti-vax movement.

The question remains, can we expect those without power and those who are being deprived to behave according to One Health approaches? In most societies, waiting in the wings, and not being able to access essential services can result in aggressive communication or even physical attack. There are differences, of course, inherent in culture to not tolerate such conduct, such as in the Scandinavian countries. In many other countries and may be in Scandinavia as well, there are risks that a pandemic like that of COVID-19 and its restrictions will continue to unlock latent resentment and anger of those less fortunate. The many implications of largely unregulated social media make it easier to develop extremist views that grow fast in times of general hardship when people look desperately for information and support, and unfortunately often encounter facile answers (3).

Addressing the fundamental question of what we can do at this time to save future generations, the following four areas can contribute to our social and scientific environments (4, 5):

(1) Identify the barriers to changing unsustainable behaviors.
(2) Employ various commitment strategies.
(3) Produce and communicate effective messages.
(4) Enhance motivation and invite participation (active civility).

Despite the promise of these strategies, limitations are also evident. The present trajectories cannot be redirected by the global North alone; effective international cooperation will be of central relevance. Physicians and veterinarians are essential professions that likewise cannot change the presently prevailing trends alone, and contributions from the social and political sciences are vital for implementing change successfully. In summary, the space between bottom-up and top-down, between grassroots and organized action needs to be filled by all of us. To progress toward One Health, it will not be enough to organize the *Connected*: we must also proactively engage impoverished and marginalized *Disconnected* populations (3) to negotiate dangerous times and unpredictable futures.

## Author contributions

All authors listed have made a substantial, direct, and intellectual contribution to the work and approved it for publication.

## Conflict of interest

The authors declare that the research was conducted in the absence of any commercial or financial relationships that could be construed as a potential conflict of interest.

## Publisher's note

All claims expressed in this article are solely those of the authors and do not necessarily represent those of their affiliated organizations, or those of the publisher, the editors and the reviewers. Any product that may be evaluated in this article, or claim that may be made by its manufacturer, is not guaranteed or endorsed by the publisher.

## Footnotes

^1^ https://www.eiu.com/n/rich-countries-will-get-access-to-coronavirus-vaccines-earlier-than-others/
